# Docbate: A National Medical Student Debate

**DOI:** 10.1007/s40596-017-0697-1

**Published:** 2017-03-17

**Authors:** Roxanne C Keynejad, Sivahamy Creed, Matthew Fernando, David Bell, David Codling, George Crowther, Judith R Harrison, Saba Jaleel, Kimberley Kendall, Lauren Megahey, Edward Noble, Claire O’Donnell, Felicity Pilcher, Tara Walker, David McLaughlan

**Affiliations:** 1 South London and Maudsley NHS Foundation Trust, London, UK; 2 South West London and St George’s Mental Health NHS Trust, London, UK; 3 Belfast Health and Social Care Trust, Belfast, UK; 4 Leeds Institute for Health Sciences, University of Leeds, Leeds, UK; 5 MRC Centre for Neuropsychiatric Genetics and Genomics, Cardiff University, Cardiff, UK; 6 Birmingham and Solihull Mental Health NHS Foundation Trust, Birmingham, UK; 7 Southern Health and Social Care Trust, Portadown, UK; 8 Worcester Health and Care NHS Trust, Worcester, UK

**Keywords:** Medical Students, Live Tweets, Medical Education Tool, Flipped Classroom, Service User Experience

Debating develops critical thinking, empathy, and verbal communication [1] but it has not been widely studied in medical education. In postgraduate teaching, debating is highly rated by learners [2–4]. It is well-suited to psychiatry as it illustrates the complex discussion and pragmatic decision-making inherent to clinical practice. However, the preparation time required may limit generalized use of debating, so implementing a single format across multiple sites might be a more efficient approach.

Social media platforms are widely used by clinicians. For example, Twitter facilitates dialogue between practitioners by sharing information, discussing research findings, and creating networks [5]. However, it is under-investigated as a medical education tool [6]. A recent systematic review found a paucity of evidence of the effectiveness of social media for teaching [7]. There is also an emphasis on challenges, rather than opportunities, in the descriptive literature [8]. Postgraduate education in psychiatry has been slower to utilize social media approaches compared to other specialties, despite evidence of benefits [9]. The limited available studies suggest that using social media in educational interventions may increase their audience and appeal to the current generation of medical students.

Here, we describe the implementation and evaluation of a pilot debating event called ‘Docbate.’ It was led by trainee psychiatrists and harnessed social media to extend its reach outside the lecture theatre. Docbate aimed to facilitate medico-political debate by engaging medical students in discussing a contentious subject. It also aimed to foster interaction between students and psychiatry trainees, by matching them in debating teams. Furthermore, Docbate aimed to challenge perceived barriers between specialties, by showing psychiatrists engaging with broad clinical questions, and to promote psychiatry through positive role modelling.

## Method

### Sites

Docbate was piloted in 2015 across six university sites: Belfast, Birmingham, Cardiff, Leeds, South-East, and South-West London. It was publicized to medical students via Facebook, Twitter, email, and on-campus posters. Docbate was a live debating event with an audience of medical students. It took place simultaneously at five sites in April. It ran in Birmingham in May, to accommodate student examinations.

### Leadership

Local trainee psychiatrists were recruited nationally through the Royal College of Psychiatrists’ Psychiatric Trainees’ Committee (PTC). They each recruited teams of medical students to local organizing committees. Trainees and students worked together to plan and promote their Docbate event via social media, as well as face-to-face promotion among peers. Each site used email advertisements to recruit two teams comprising one student and one doctor each.

### Debates

Twelve third year medical students took part in a focus group, led by supervising author DM. The students were asked what was most exciting and most controversial about psychiatry. They complied a shortlist of possible motions for the debate. Members of the focus group, the PTC, and principal funder voted on the shortlist. The motion “Cash, care and coercion: doctors do know best” was ultimately selected as having greatest potential for balanced debate. During Docbate, each team member spoke alternately, for or against the motion.

### Panel Discussions

After the debate, a panel comprising invited academics, journalists, service users, and advocates discussed the issues raised, with audience participation. At each site, the content of debate varied but included discussion of patient rights, deprivation of liberty, clinical autonomy, and public health decisions. Panel discussions linked these issues to psychiatry, particularly service user experiences, and the ethical and medico-legal aspects of clinical practice. Points of discussion, audience questions, and vote results were live-tweeted by organizers and attendees: this fostered debate during and after the event.

### Logistics

The event was free to attend. The mean cost per site was £460 for refreshments and promotional material. The universities waived venue hire. Attendees were informed that some organizers, debaters, and panelists were psychiatrists. Funding from the Royal College of Psychiatrists was acknowledged.

### Evaluation

Medical student attendees were invited to complete questionnaires before and after Docbate. The questionnaire included demographic details and free text boxes probing positive aspects and suggested improvements.

## Results

One hundred and sixty-three people attended docbate; 93 questionnaires were completed by medical students across six sites, of whom 57 (61%) were female. The audience ranged in age from 18 to 52 years, with a mean age of 26 years and 3 months.

Thirty percent of attendees heard about Docbate online. Docbate.com received 2382 unique visitors and 7935 page views. Docbate was mentioned in 520 tweets between 26 April 2015 and 26 May 15. The event was reported in a British Medical Association News bulletin sent to 170,000 UK doctors [10].

Ninety-eight percent enjoyed Docbate, 94% would recommend it to a friend, and 94% would attend in the future. Qualitative feedback indicated that some attendees gained new knowledge (for example, that the UK government had proposed to pay general practitioners a fee for every case of dementia diagnosed). However, attendees more commonly praised the opportunity to observe and engage in the debate (42 respondents). Additionally, they specifically praised the panelists (27), the organization and structure of the event (9), live tweeting (8), and audience participation (8). They called Docbate “enlightening,” “thought-provoking,” “informative,” “stimulating,” and “eye-opening.” One said: “I have learnt a lot about the structure and funding of the NHS (National Health Service). I did not appreciate the importance of this and the effect it has on doctors’ everyday practice.” When asked for suggested improvements, students recommended better publicity or timing in the academic year to ensure a larger audience (17), narrowing the motion and defining terms to focus the debate (15), better time-keeping (6), and greater audience participation (5).

## Discussion

This pilot debating event reached a range of stakeholders, including the general public, service users, students, and doctors. The cost of the event was relatively modest and Docbate expanded its reach through social media. Fifty-seven percent of attendees completed questionnaires but it is unclear how many of the remaining 43% were medical students. The debate, panel, interactive discussion, and live tweeting were positively received and Docbate was rated highly by attendees. Suggested improvements included better publicity for Docbate, timing to accommodate examinations, and a clearer and narrower motion. Respondents rated a number of aspects of Docbate positively, making it difficult to determine whether the debate, panel discussion, social media use, or a combination had the greatest impact. It is also likely that the event attracted a subset of students with a prior interest in psychiatry.

Future studies of this method should include control groups, who were not exposed to any additional teaching, or who attended teaching on the same subject by an established method, such as a lecture or flipped classroom. Longitudinal follow-up could assess whether Docbate prompted interest in new areas of study and what students learned after participating. Future events should encourage all present to complete questionnaires, to monitor attendance demographics. Online surveys could be completed by those who engaged with the event remotely. The scope of Docbate was limited by its one-off nature and the choice of a motion, which did not address psychiatry explicitly. Live streaming, follow-up blogs, podcasts, and a wider e-learning program could expand this model’s influence beyond a single event. In order to attract students who are not already interested in psychiatry, future iterations of Docbate should collaborate with a range of student societies and postgraduate associations, bridging gaps between specialties. Social media evolves continuously: this event must adapt to remain attractive, accessible, and relevant to new cohorts of medical students.

Although still in its infancy, the use of social media in psychiatric education has potential to facilitate international and cross-cultural communities of practice. For example, trainees at the Johns Hopkins University School of Medicine discuss seminal psychiatric works with peers at the South London and Maudsley NHS Foundation Trust in the UK, asynchronously [11]. Medical students in Somaliland unable to access face-to-face mental health education have rated synchronous online peer discussions highly [12]. The growing availability of internet and smartphone technology means that creative e-learning adaptations of traditional debating methods have the potential to expand the medical school classroom across the nation and the globe.
